# Associations between physical activity patterns and dietary patterns in a representative sample of Polish girls aged 13-21 years: a cross-sectional study (GEBaHealth Project)

**DOI:** 10.1186/s12889-016-3367-4

**Published:** 2016-08-02

**Authors:** Lidia Wadolowska, Joanna Kowalkowska, Marta Lonnie, Jolanta Czarnocinska, Marzena Jezewska-Zychowicz, Ewa Babicz-Zielinska

**Affiliations:** 1 Department of Human Nutrition, University of Warmia and Mazury in Olsztyn, Sloneczna 45F, 10-718 Olsztyn, Poland; 2 Department of Human Nutrition and Hygiene, Poznan University of Life Sciences, Wojska Polskiego 28, 60-637 Poznan, Poland; 3 Department of Organisation and Consumption Economics, Warsaw University of Life Sciences, Nowoursynowska 159 C, 02-776 Warsaw, Poland; 4 Department of Trade and Services, Gdynia Maritime University, Morska 81-87, 81-225 Gdynia, Poland

**Keywords:** Active recreation, Adolescents, Dietary patterns, Girls, Physical activity, Principal Component Analysis, School activity

## Abstract

**Background:**

Similar to other countries, trends of decreasing levels of physical activity (PA) and an increasing prevalence of unhealthy dietary patterns are observed among girls in Poland. Better understanding of potentially inter-related behaviours within this population can help to design tailored interventions. The purpose of this study was to determine associations between PA patterns and dietary patterns in a representative sample of Polish girls.

**Methods:**

Girls aged 13-21 years (*n* = 1107) were randomly selected for the study. PA was assessed using International Physical Activity Questionnaire – Long (IPAQ-L). Dietary data were collected with food frequency questionnaires. PA patterns and dietary patterns were drawn separately by Principal Component Analysis (PCA). Logistic regression was used to find the associations between PA patterns and dietary patterns.

**Results:**

Four major PA patterns (‘School/work activity’, ‘Active recreation’, ‘Yard activity’ and ‘Walking and domestic activity’) and four dietary patterns (‘Traditional Polish’, ‘Fruit & vegetables’, ‘Fast food & sweets’ and ‘Dairy & fats’) were identified. Level of PA was the highest in the upper tertile of ‘School/work activity’ pattern (mean 1372.2 MET-minutes/week, 95 % Confidence Intervals [CI]: 1285.9–1458.5). Girls in upper tertiles of ‘Yard activity’, ‘Active recreation’ and ‘School/work activity’ patterns had significantly higher chances of being in the upper tertile of the ‘Fruit and vegetables’ dietary pattern (odds ratio [OR] 2.17, 95 % CI: 1.50–3.14, *p* < 0.0001; OR 2.02, 95 % CI: 1.41–2.91; *p* < 0.001 and OR 1.76, 95 % CI: 1.24–2.51, *p* < 0.01 respectively; all adjusted for confounders) in comparison to bottom tertiles. Weak, but significant inverse associations were found between upper tertiles of ‘Active recreation’ and ‘Yard activity’ patterns and unhealthy dietary patterns.

**Conclusions:**

We found associations between PA patterns and dietary patterns in the population of Polish girls. Girls with the highest adherence to the ‘School/work activity’ pattern had the highest levels of PA and presented pro-healthy dietary behaviours. School should be recognised as potentially efficient and important setting to maximise girls' PA potential. The after-school time is the area that should also be targeted to increase daily PA or to at least sustain the level of PA after completing education.

**Electronic supplementary material:**

The online version of this article (doi:10.1186/s12889-016-3367-4) contains supplementary material, which is available to authorized users.

## Background

Adequate levels of physical activity combined with a balanced diet are two essential components of healthy lifestyle and are main factors in obesity prevention [1]. Maintaining healthy weight improves self-esteem in adolescents [2, 3] and decreases the risk of developing chronic diseases in later life [4, 5]. Despite the health benefits, the decline in levels of physical activity as young people age is observed worldwide [6, 7], including Poland where only 15.2 % of girls aged 11–17 reach adequate levels of physical activity recommended by World Health Organisation [8]. Furthermore, the ‘westernisation’ of Polish culture is also reflected in changes in Polish adolescents’ diets. A shift from Polish staple foods towards highly processed, high-fat, high-sugar and low-fibre foods became more apparent over the last two decades and is becoming a matter of public health concern which needs to be addressed [9].

Better understanding of health-related behaviours and the potential associations between them can help to design interventions [10, 11]. At present it is unclear if the behaviours share a common determinant and whether interventions should target physical activity and nutrition separately or simultaneously to maximise its effectiveness.

In youth, physical activity and dietary behaviours are complex and it is argued whether underlying mechanisms (e.g., driven by personality traits or health concerns) of health related-behaviours exist in this population [12–14]. In adult populations, people who are more active tend to have healthier diets [15, 16]. The most often reported motives in their case are to be fit or simply to be healthy [17, 18]. In contrary, young females are rarely concerned about health [19] and present more hedonistic attitudes towards life [20]. Studies which conducted focus groups revealed that ‘enjoyment’ tend to be more important in terms of physical activity participation [21] and ‘liking’ or ‘preferences’ are stronger food choice drives than expected health outcomes [20, 22]. This indicates that young people may engage in a mixture of different behaviours (healthy/unhealthy) and a more holistic approach is needed to evaluate which behaviours tend to cluster and which do not; if the clustering exists, what is the potential explanation?

Recent approaches in studying clustering of health-related behaviours have been to identify the patterns of behaviours rather than investigating individual exposures, i.e., particular foods intakes [23, 24]. One of the statistical methods used in examining behavioural patterns is Principal Component Analysis (PCA). PCA allows analysis of how different types of behaviours correlate in a given population [25]. Studies of adolescents’ dietary patterns which used PCA have provided a valuable insight into gender and age-related differences in terms of young peoples’ food choices [26–28]. In general, girls tend to make healthier food choices than boys [26–28] and overall diet quality declines with age despite gender [29].

Although the studying of dietary patterns has recently gained a lot of interest, only a limited number of studies tried to describe the patterning of physical activity in girls [30]. Furthermore, fewer studies investigated the associations between dietary patterns and physical activity in adolescent girls, with results remaining inconsistent [14, 31–35]. For example, Pearson et al. [34] found that unhealthy dietary behaviours were clustering with low levels of physical activity, while Ottevaere et al. [31] found two clusters in the studied population that would appear to be contradictory, i.e., ‘active, low quality diet’ and ‘inactive, high quality diet’. The inconsistencies in previous studies may have been a result of using hypothesis-driven (*a priori*) approach which is based on existing evidence and prior knowledge [24]. For example, dietary patterns were examined in relation to pre-defined exposures, such as sedentary behaviour or screen time rather than the patterning of activities, either proving or disapproving the hypothesis [14]. In contrast to previous studies we have applied an exploratory approach and identified the two groups of patters using statistical methods [24]. We believed that using this data-driven *(a posteriori)* approach in finding the associations between diet and physical activity can bring some novel findings, which may not have been examined before.

We decided to focus on girls for two reasons. Firstly, girls in general are less active than boys and the decline in physical activity as they age is more profound in comparison to boys, indicating it might be a population at higher risk [34, 35]. Secondly, with emerging evidence about the role of early life nutritional exposures on future health [36] and maternal modelling [37], the population of females of pre- and reproductive age is of a particular concern. The participants of our study included girls aged 13–21 years and consisted of both ‘adolescents’ and ‘youth’ [38]. However, for simplicity, the study population will be referred to as ‘girls’ throughout the paper.

### Objective

In our study we hypothesised that girls who present particular physical activity patterns may also present specific patterns of dietary behaviours. Thus, the aim of our study was twofold: 1) to empirically identify physical activity patterns and dietary patterns in a representative sample of Polish girls using PCA, and 2) if the patterns were found, to investigate the associations between physical activity patterns and dietary patterns.

## Methods

### Data collection

Data came from the GEBaHealth (Girls Eating Behaviours and Health) project; a cross-sectional study regarding diet, attitudes towards food, nutrition and health as well as physical activity and obesity in a representative sample of Polish girls. A closed-question questionnaire was used. The study was conducted in person by trained interviewers at respondents’ home. Recruitment, data collection and entry were made by the Public Opinion Research Centre (CBOS, Warsaw, Poland). All data were collected in 2012.

### Participants

**Fig. 1:**
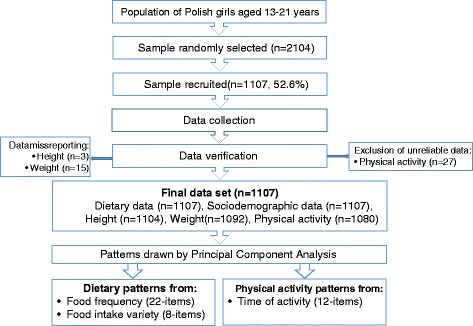
Flowchart: Study design and data collection

Details of the study design and sample collection were described previously [39]. Briefly, the sample was randomly selected using three-phase sampling from females born from 1991 to 1999 and living in Poland (Fig. 1), which were the criteria for inclusion in the study. Sample size calculation was based on our previous pilot study. Means were obtained for 42 variables: food frequency consumption (22 items), food intake variety (8 items) and physical activity (12 items). Assuming 5 and 10 % relative error and 95 % confidence interval (CI) a minimum sample size was initially calculated for each variable separately. Taking into account the median of a minimum sample size for all variables, the overall minimum sample size of 1029 (for 5 % relative error) and 257 (for 10 % relative error) was calculated. Therefore, it was decided that a minimum sample size of roughly 1000 participants was needed. Next, considering the estimated rates given by CBOS (rate for design effect and non-response), a total of 2104 girls aged 13–21 years were chosen by date of birth, using PESEL number (Universal Electronic System of Population Register). The response rate was 52.6 %. The main reasons of missing data were: respondents' absence or parent/guardian/respondent’s refusal. Finally, the study was carried out in 1107 girls. Sample weights were applied to obtain nationally representative data, i.e., to adjust for unequal selection and non-response. The weighting variables were age (three categories), place of residence (rural/urban) and country’s regions.

### Measurements of physical activity

A validated International Physical Activity Questionnaire (IPAQ-L), long form, in Polish language was used [40]. For 12-items of physical activity, the duration (in minutes) and frequency (days) for various types of activities in four domains were collected: leisure time, domestic and gardening, school- or work-related and transport-related. Activities during the last 7 days were considered. Unreliable reports of physical activity were identified in 27 participants (mean weekly time of activity >960 min/day) and therefore these data (not participants) were removed. The physical activity level was expressed as a standard Metabolic Energy Turnover (MET) in MET-minutes/week according to the procedure prepared by the IPAQ Research Committee [40]. Each item of activity had a corresponding MET value assigned, e.g., walking = 3.3, moderate activity = 4.0, cycling = 6.0 and vigorous activity = 8.0. For each item of activity, MET-minutes/week scores were computed by multiplying the MET value by the time (minutes and days) spent on these activities for each participant. For example, the formula for computation of MET-minutes/week spent on cycling was as follows: cycle MET-minutes/week for transport = 6.0*cycling minutes*cycle days for transportation. An overall total physical activity (in MET-minutes/week) was calculated as a sum of the scores of all the activities. According to IPAQ scoring protocol [40], the categorical scores to define the level of physical activity as low, moderate or high were <600 MET-minutes/week, 600–2999 MET-minutes/week and ≥3000 MET-minutes/week, respectively.

### Measurements of dietary characteristics

A food frequency method was applied. Details of dietary data collection were described previously [39]. Briefly, three short validated food frequency questionnaires were used: Block Screening Questionnaire for Fruit/Vegetable/Fibre Intake (BSQFVF) [41], Block Screening Questionnaire for Fat Intake (BSQF) [41] and Food Intake Variety Questionnaire (FIVeQ) [42]. We used both Block questionnaires after modification and adjustment to a typical Polish diet and language [39]. In total, 22 food items from Block questionnaires were considered; consumption frequency was expressed in points in five categories:

1. **BSQFVF**: less than once per week (0 points), about once per week (1 point), 2–3 times per week (2 points), 4–6 times per week (3 points), daily (4 points) – for 9 food items: fruit or vegetable juices, fruit (without juices), green salad, potatoes, beans, prepared vegetables (e.g., cooked, preserved or marinated, excluding beans), high-fibre or bran cereal, wholegrain bread, white bread (including biscuits, muffins).
2. **BSQF**: less than once per month (0 points), 2–3 times per month (1 point), 1–2 times per week (2 points), 3–4 times per week (3 points), 5 times per week and more (4 points) – for 13 food items: hamburgers or cheeseburgers, red meats (e.g., pork, beef), fried chicken, hot dogs or frankfurters, luncheon meats or bacon or fatty sausages, salad dressings or mayonnaise (not diet), margarine or butter, eggs, cheese or cheese spread, whole milk, French fries or potato chips or corn chips or popcorn, ice cream, doughnuts or pastries or cake or cookies.

Food intake variety assessed with FIVeQ was measured for 60 foods from 8 food groups: cereals and potatoes (6 items), dairy products (4 items), meats, fish and eggs (12 items), vegetables (14 items), fruit (8 items), fats (6 items), sweets and snacks (4 items), beverages (6 items, without alcohol) [42]. The frequency of consumption was collected in two categories (yes/no). Answer ‘yes’ concerned frequency of food consumption after thermal treatment (“ready to eat”), during the last 7 days, in an amount usually greater than 2 tablespoons or 7 bread slices or 7 glasses. ‘Yes’ answers were summed up for each food group separately and food intake variety was expressed as a number of food items consumed weekly (foods/week).

### Confounding factors

Socioeconomic variables were collected using standard questions and coded as numerical data (in points) as follows: father’s and mother’s education – primary/lower secondary (1), upper secondary (2), higher (3); residence – village (1), town (2), city (3); self-declared economic situation – below average (1), average (2), above average (3). Socioeconomic status (SES) index was created for description of overall socio-economic status. Details regarding the SES index were described previously [39]. Briefly, the SES index was calculated as the sum of numerical values assigned to each category of single socioeconomic variables. The assigned categories of SES index were based on tertile distribution.

Participants were asked about their weight and height. Self-reported height and weight were corrected using regression equations [43] and Body Mass Index (BMI) was calculated.

Weight status categories for all study participants were assigned according to the revised International Obesity Task Force (IOTF) standards [44]: thinnest grade 3 (BMI < 16.0 kg/m^2^), thinnest grade 2 (BMI = 16.0–16.9 kg/m^2^), thinnest grade 1 (BMI = 17.0–18.4 kg/m^2^), normal weight (BMI = 18.5–24.9 kg/m^2^), overweight (BMI = 25.0–29.9 kg/m^2^) and obesity (BMI ≥ 30 kg/m^2^). For girls 13–18 years old BMI age-sex-specific BMI values corresponding to the cut-offs at age 18 were applied. For girls 18–21 years old BMI cut-offs at age 18 (for adults) were used [44].

### Statistical analysis

Physical activity patterns and dietary patterns were separately derived by the Principal Component Analysis (PCA). Various combinations of variables were checked. Before analysis input variables were standardised. The factorability of data was confirmed with Kaiser-Meyer-Olkin (KMO) measure of sampling adequacy greater than 0.5 and Bartlett’s test of sphericity achieving statistical significance [45]. KMO value for physical activity data was 0.631 and Bartlett’s test had a significance of *p* < 0.0001. KMO value for dietary data was 0.776 and Bartlett’s test had a significance of *p* < 0.001. To derive both groups of patterns a varimax normalised rotation was used in order to extract non-correlated factors and obtain large variance explained [45]. Eigenvalues of at least 1.00 were considered. To derive physical activity patterns the input variables were 12-items of the duration of physical activities (in minutes). The physical activities with factor-loadings of at least 0.50 have been considered and used to label the physical activity patterns. As most of the girls were studying, after physical activity patterns were derived (for 1107 girls), additional PCA was run with the exclusion of 47 girls solely in employment (Additional file 1: Table S1). Since the physical activity patterns (for 1060 girls) were very similar it was decided not to remove any participants from the analysis to ensure the representativeness of the national data (Additional file 2: Table S2). To derive dietary patterns the input variables were 22-items of food frequency consumption (from BSQFVF and BSQF) and 8-items of food intake variety (from FIVeQ); all in points. Dietary characteristics with factor-loadings of at least 0.40 have been considered and used to label dietary patterns. The lower cut-off for factor-loading to derive dietary patterns was used because of greater variability of dietary characteristics than physical activities. Based on tertiles distribution participants were divided into three categories within each pattern (bottom, middle, upper tertile), separately for physical activity patterns and dietary patterns.

The associations between physical activity patterns and dietary patterns were verified by a logistic regression analysis. The odds ratios (ORs) and 95 % CI were calculated. Odds ratios represented the associations between girls in middle or upper tertiles of dietary patterns and the adherence to middle or upper tertiles of physical activity patterns. The reference groups were participants from bottom tertiles of physical activity patterns (OR = 1) and dietary patterns (OR = 1.00).

The significance of ORs was assessed by Wald’s statistics. Two models were created: (i) unadjusted – without adjustment for confounding factors and (ii) adjusted for age and SES index (as continuous variables) and BMI (as a categorical variable).

Variables normality was checked by Kolmogorov-Smirnov test. Continuous data are presented as means with 95 % CIs. The differences between groups were verified by 2-tailed *t*-test [45]. All data were logarithmically transformed before analysis. All analyses were conducted with sample weights to correct non-response data during the sample collection. For all tests, *p*-value <0.05 was considered as significant. The statistical analysis was carried out using STATISTICA software (version 10.0 PL; StatSoft Inc., USA, Tulsa; StatSoft Polska, Krakow).

## Results

### Sample characteristics

Table 1 displays characteristics of the study sample. The mean age of participants was 17.3 years (95 % CI: 17.1–17.4). Based on tertile distribution 36.2 % of girls had low SES index, 30.6 % medium and 33.2 % high. Self-reported weight and height were obtained from 1092 participants. The majority of the study sample (77.7 %) were classified as normal weight, 10.2 % were underweight, 10.5 % overweight and 1.6 % were classified as obese. Only one girl from the study sample had a BMI ≥ 35 kg/m^2^ (morbid obesity) and therefore the two weight status categories (obesity and morbid obesity) were combined into one category – obesity. According to IPAQ scoring protocol [40], 47.1 % of girls had physical activity level classified as low (<600 MET-minutes/week), 50.9 % were classified as moderate (600–2999 MET-minutes/week), and only 2 % of girls met the high physical activity level of ≥3000 MET-minutes/week.

**Table 1:** Sample characteristics

		Total sample
Number of participants		1107
Gender	Female (%)	1107 (100.0)
Age (years)	Mean (95 % Confidence interval)	17.3 (17.1; 17.4)
	Minimum – maximum	13–21
SES Index ^a^	Low	401 (36.2)
	Medium	339 (30.6)
	High	367 (33.2)
Mother’s education	Primary/lower secondary (%)	441 (39.8)
	Upper secondary (%)	484 (43.8)
	Higher (%)	182 (16.5)
Father’s education	Primary/lower secondary (%)	579 (52.4)
	Upper secondary (%)	389 (35.1)
	Higher (%)	139 (12.5)
Residence	Country (%)	521 (47.1)
	Town (%)	348 (31.4)
	City (%)	238 (21.5)
Economic status	Below average (%)	44 (3.9)
	Average (%)	885 (80.0)
	Above average (%)	178 (16.1)
BMI category ^b^	Thinnest grade 3 (%)	0 (0)
	Thinnest grade 2 (%)	5 (0.5)
	Thinnest grade 1 (%)	105 (9.7)
	Normal weight (%)	849 (77.7)
	Overweight (%)	115 (10.5)
	Obesity	18 (1.6)
Physical activity ^c^	Low (%)	509 (47.1)
	Moderate (%)	550 (50.9)
	High (%)	21 (2.0)

Sample size may vary in each variables due to missing data. All data adjusted for sample weights

^a^ SES index categories based on tertile distribution

^b^ BMI: body mass index (*n* = 1092); Weight status categories assigned according to IOTF standards [44]; for girls 13–18 years old BMI age-sex-specific cut-offs were corresponding to the values at age 18; for girls >18 years old according to cut-offs for girls at age 18 (adults)

^c^ Physical activity classification: low: <600 MET-minutes/week, moderate: 600–2999 MET-minutes/week, high: ≥3000 MET-minutes/week, according to IPAQ protocol [40]

95 % CI (Confidence Interval)

### Physical activity patterns

Four physical activity patterns were found: ‘School/work activity’, ‘Active recreation’, ‘Yard activity’ and ‘Walking & domestic activity’ (Table 2).

**Table 2:** Factor-loading matrix for the 4 major physical activity patterns identified by principal component analysis

Type of physical activity	Factor 1	Factor 2	Factor 3	Factor 4
	‘School/work activity’	‘Active recreation’	‘Yard activity’	‘Walking & domestic activity’
School/work – moderate activity	0.75			
School/work – walking	0.73			
School/work – vigorous activity	0.72			
Leisure-time – moderate activity		0.80		
Leisure-time – vigorous activity		0.73		
Active transportation – cycle		0.61		
Yard work – vigorous activity			0.76	
Yard work – moderate activity			0.79	
Active transportation – walking				0.73
Leisure-time – walking				0.61
Home activity – moderate				0.50
Sitting				
Factor intercorrelations				
Factor 1 (School/work activity)	–			
Factor 2 (Active recreation)	0.13	–		
Factor 3 (Yard activity)	0.10	0.13	–	
Factor 4 (Walking & domestic activity)	0.12	0.06	0.11	–
Eigenvalues	2.07	1.51	1.43	1.25
Variance explained (%)^b^	17.3	12.6	11.9	10.4

Factor loadings of ≤ |0.50| are not shown in the table for simplicity. Sorted by loadings from 1^st^ to 4^th^ factor. All data adjusted for sample weights

^a^ Physical activity was expressed in MET-minutes/week

^b^ Total variance in physical activity variables explained by 4 patterns is 52.2 %

Total variance explained by four patterns was 52.1 %. For each pattern variance explained was: 17.3 % (‘School/work activity’), 12.6 % (‘Active recreation’), 11.9 % (‘Yard activity’) and 10.4 % (‘Walking & domestic activity’). Factor intercorrelations were very weak ranging from *r* = 0.06 to *r* = 0.13 [46]. The ‘School/work activity’ pattern was described by time spent with various levels of activity at school or work: moderate (factor-loading 0.75), walking (0.73), vigorous (0.72). The ‘Active recreation’ pattern was described by time spent with two levels of activities within leisure-time: moderate (0.80) and vigorous (0.73), and time spent with active transportation by cycling (0.61). The ‘Yard activity’ pattern was described by time spent with two levels of yard maintenance activities (e.g., shoveling snow, carrying loads or gardening): vigorous (0.76) and moderate (0.79). The ‘Walking & domestic activity’ pattern was described by time spent walking while active transportation (0.73) and leisure-time (0.61), time spent with moderate activity at home (0.50) and time spent sitting (0.47) (Table 2). In upper tertiles of all physical activity patterns, the highest level of physical activity was found in the ‘School/work activity’ pattern (mean 1372.2 MET-minutes/week, 95 % CI: 1285.9–1458.5) and the lowest in the ‘Yard activity’ pattern (mean 1053.1, 95 % CI: 984.5–1121.6) (Table 3).

**Table 3:** Mean physical activity in MET-minutes/week (with 95 % Confidence Intervals) by tertiles of physical activity patterns

		Bottom	Middle	Upper
Total physical activity		284.5^a,b^ (273.3; 295.7)	626.6^a,c^ (615.4; 637.9)	1555.8^b,c^ (1478.7; 1632.9)
‘School/work activity’ pattern	A	505.6^a,b^ (459.6; 551.6)	608.0^a,c^ (574.4; 641.6)	1372.2^b,c^ (1285.9; 1458.5)
‘Active recreation’ pattern	B	822.8^a,b^ (742.6; 903.0)	596.1^a,c^ (548.0; 644.3)	1076.2^b,c^ (1000.4; 1152.1)
‘Yard activity’ pattern	C	818.9^a,b^ (736.3; 901.4)	625.1^a,c^ (567.2; 682.9)	1053.1^b,c^ (984.5; 1121.6)
‘Walking & domestic activity’ pattern	D	678.4^a^ (608.7; 748.0)	702.4^b^ (641.6; 763.2)	1113.7^a,b^ (1036.8; 1190.6)
		A-B, A-C, A-D, B-D, C-D	A-D, B-D	A-B, A-C, A-D

All data adjusted for sample weights and logarithmically transformed before analysis

a-a, b-b, c-c – significant differences between tertiles within each pattern at *p* < 0.05

A-B,…, C-D – significant differences between the same tertiles of various patterns at *p* < 0.05

### Dietary patterns

Four dietary patterns were found: ‘Traditional Polish’, ‘Fruit & vegetables’, ‘Fast food & sweets’ and ‘Dairy & fats’ (Table 4).

**Table 4:** Factor-loading matrix for the 4 major dietary patterns identified by principal component analysis

		Factor 1	Factor 2	Factor 3	Factor 4
		‘Traditional Polish’	‘Fruit & vegetables’	‘Fast food & sweets’	‘Dairy & fats’
Food frequency consumption of^a^:	White bread (including biscuits, muffins)	0.65			
	Potatoes	0.52			
	Red meats	0.51			
	Margarine or butter	0.45			0.45
	Fried chicken	0.42			
	Wholegrain bread	−0.48			
	Green salad		0.57		
	Fruit (without juices)		0.55		
	Prepared vegetables		0.55		
	Beans		0.45		
	French fries or potato chips or corn chips or popcorn			0.71	
	Hamburgers or cheeseburgers			0.60	
	Ice cream			0.52	
	Doughnuts or pastries or cake or cookies			0.50	
	Salad dressings or mayonnaise (not diet)			0.42	
	Cheese or cheese spread				0.54
	Whole milk				0.49
Food intake variety by food groups^b^:	Meats, fish and eggs	0.60			
	Fats	0.45			0.43
	Vegetables		0.60		
	Fruit		0.54		
	Sweets and snacks			0.47	
	Cereals and potatoes				0.56
	Dairy products				0.54
Factor intercorrelations	Factor 1 (Traditional Polish)	–			
	Factor 2 (Fruit & vegetables)	0.14	–		
	Factor 3 (Fast foods & sweets)	0.44	0.13	–	
	Factor 4 (Dairy and fats)	0.34	0.41	0.39	–
Eigenvalues		4.36	2.39	1.68	1.44
Variance explained (%)^c^		14.5	9.0	5.6	4.8

Factor loadings of ≤ |0.40| are not shown in the table for simplicity. Sorted by loadings from 1^st^ to 4^th^ factor. All data adjusted for sample weights

^a^ Food frequency consumption was expressed in points (range 0–4 points)

^b^ Food intake variety was expressed in foods consumed per week (with ranges from 0–4 to 0–14 foods/week)

^c^ Total variance in dietary variables explained by 4 patterns is 33.9 %

Total variance explained was 33.9 %. For each pattern variance explained was: 14.5 % (‘Traditional Polish’), 9.0 % (‘Fruit & vegetables’), 5.6 % (‘Fast food & sweets’) and 4.8 % (‘Dairy & fats’). Factor intercorrelations were very weak to moderate, ranging from *r* = 0.13 to *r* = 0.44 [46]. The ‘Traditional Polish’ pattern was described by frequent consumption of: white bread (factor-loading 0.65), potatoes (0.52), red meats (0.51), margarine or butter (0.45), fried chicken (0.42), wholegrain bread (-0.48; the reverse relation) and also food intake variety of meats/fish/eggs (0.60), fats (0.40). The ‘Vegetables & fruit’ pattern was described by frequent consumption of: green salad (0.57), fruit (0.55), prepared vegetables (0.55), beans (0.45), and also food intake variety of vegetables (0.60) and fruit (0.54). The ‘Fast food & sweets’ pattern was described by frequent consumption of: French fries or potato chips or corn chips or popcorn (0.71), hamburgers or cheeseburgers (0.60), ice cream (0.52), doughnuts or pastries or cake or cookies (0.50), salad dressings or mayonnaise (not diet) (0.42), and also food intake variety of sweets and snacks (0.47). The ‘Dairy & fats’ pattern was described by frequent consumption of: cheese or cheese spread (0.54), whole milk (0.49), margarine or butter (0.45), and food intake variety of cereals and potatoes (0.56), dairy products (0.54), and fats (0.43). Additional data regarding dietary characteristics and physical activities are shown in Additional file 3: Table S3.

### Associations between physical activity patterns and dietary patterns

Girls in the upper tertile of ‘School/work activity’ pattern were more likely to fall in the upper tertile of ‘Fruit & vegetables’ pattern (Odds Ratio [OR] 1.76, 95 % CI: 1.24–2.51, after adjustment for age, SES and BMI) in comparison to the bottom tertile (Table 5). Girls in the upper tertile of ‘Active recreation’ pattern were more likely to fall in the upper tertile of ‘Fruit & vegetables’ pattern (adjusted OR 2.02, 95 % CI: 1.41–2.91) and less likely to fall in the upper tertile of ‘Traditional Polish’ pattern (adjusted OR 0.52, 95 % CI: 0.36–0.75) in comparison to bottom tertiles. Girls in the upper tertile of ‘Yard activity’ pattern were more likely to fall in the upper tertile of ‘Fruit & vegetables’ pattern (adjusted OR 2.17, 95 % CI: 1.50–3.14) and less likely to fall the in upper tertile of the ‘Fast food & sweets’ pattern (adjusted OR 0.53, 95 % CI: 0.37–0.76) in comparison to bottom tertiles. The ‘Walking & domestic activity’ pattern was not associated with any dietary patterns, with the exception of the middle tertile of the ‘Traditional Polish’ pattern. Girls in the upper tertile of total physical activity were more likely to fall in the upper tertile of the ‘Fruit and vegetables’ pattern (adjusted OR 2.47, 95 % CI: 1.73–3.54) in comparison to the bottom tertile of total physical activity. Unadjusted associations between physical activity patterns and dietary patterns can be found in Additional file 4: Table S4.

**Table 5:** Adjusted associations between physical activity patterns and dietary patterns (Adjusted Odds Ratios with 95 % Confidence Intervals)

Physical activity patterns	Dietary patterns	Tertiles of dietary patterns	Tertiles of physical activity patterns				
			Bottom	Middle		Upper	
‘School/work activity’	‘Traditional Polish’	Bottom	ref.	ref.		ref.	
		Middle	ref.	1.01	(0.70; 1.46)	0.98	(0.69; 1.40)
		Upper	ref.	1.03	(0.72; 1.48)	1.08	(0.75; 1.54)
	‘Fruit & vegetables’	Bottom	ref.	ref.		ref.	
		Middle	ref.	1.27	(0.89; 1.81)	1.23	(0.87; 1.75)
		Upper	ref.	1.51*	(1.04; 2.18)	1.76**	(1.24; 2.51)
	‘Fast food & sweets’	Bottom	ref.	ref.		ref.	
		Middle	ref.	1.20	(0.84; 1.72)	0.82	(0.57; 1.17)
		Upper	ref.	1.20	(0.84; 1.72)	1.04	(0.74; 1.47)
	‘Dairy & fats’	Bottom	ref.	ref.		ref.	
		Middle	ref.	1.07	(0.74; 1.53)	1.09	(0.77; 1.54)
		Upper	ref.	1.58*	(1.10; 2.26)	1.20	(0.85; 1.71)
‘Active recreation’	‘Traditional Polish’	Bottom	ref.	ref.		ref.	
		Middle	ref.	0.60**	(0.42; 0.86)	0.60**	(0.42; 0.86)
		Upper	ref.	0.64*	(0.44; 0.92)	0.52***	(0.36; 0.75)
	‘Fruit & vegetables’	Bottom	ref.	ref.		ref.	
		Middle	ref.	1.50*	(1.07; 2.11)	1.94***	(1.35; 2.78)
		Upper	ref.	1.25	(0.88; 1.77)	2.02***	(1.41; 2.91)
	‘Fast food & sweets’	Bottom	ref.	ref.		ref.	
		Middle	ref.	1.20	(0.84; 1.70)	0.90	(0.63; 1.29)
		Upper	ref.	0.85	(0.60; 1.20)	0.74	(0.53; 1.05)
	‘Dairy & fats’	Bottom	ref.	ref.		ref.	
		Middle	ref.	0.95	(0.67; 1.34)	1.12	(0.78; 1.60)
		Upper	ref.	1.12	(0.79; 1.58)	1.30	(0.91; 1.85)
‘Yard activity’	‘Traditional Polish’	Bottom	ref.	ref.		ref.	
		Middle	ref.	0.76	(0.54; 1.07)	1.02	(0.71; 1.47)
		Upper	ref.	0.83	(0.59; 1.19)	0.99	(0.69; 1.43)
	‘Fruit & vegetables’	Bottom	ref.	ref.		ref.	
		Middle	ref.	1.19	(0.85; 1.67)	1.65**	(1.15; 2.36)
		Upper	ref.	1.36	(0.96; 1.92)	2.17****	(1.50; 3.14)
	‘Fast food & sweets’	Bottom	ref.	ref.		ref.	
		Middle	ref.	0.69*	(0.49; 0.99)	0.73	(0.51; 1.06)
		Upper	ref.	0.59**	(0.41; 0.83)	0.53***	(0.37; 0.76)
	‘Dairy & fats’	Bottom	ref.	ref.		ref.	
		Middle	ref.	0.88	(0.62; 1.24)	1.07	(0.75; 1.54)
		Upper	ref.	1.14	(0.81; 1.61)	1.32	(0.92; 1.89)
‘Walking & domestic activity’	‘Traditional Polish’	Bottom	ref.	ref.		ref.	
		Middle	ref.	1.41*	(1.00; 1.99)	1.37	(0.97; 1.93)
		Upper	ref.	1.34	(0.94; 1.91)	1.34	(0.95; 1.91)
	‘Fruit & vegetables’	Bottom	ref.	ref.		ref.	
		Middle	ref.	0.86	(0.61; 1.21)	0.88	(0.62; 1.23)
		Upper	ref.	0.94	(0.67; 1.33)	0.82	(0.58; 1.17)
	‘Fast food & sweets’	Bottom	ref.	ref.		ref.	
		Middle	ref.	1.27	(0.90; 1.80)	1.02	(0.72; 1.45)
		Upper	ref.	1.26	(0.89; 1.78)	0.95	(0.68; 1.34)
	‘Dairy & fats’	Bottom	ref.	ref.		ref.	
		Middle	ref.	0.85	(0.60; 1.22)	1.01	(0.69; 1.48)
		Upper	ref.	0.93	(0.66; 1.31)	1.02	(0.72; 1.43)
Total physical activity	‘Traditional Polish’	Bottom	ref.	ref.		ref.	
		Middle	ref.	1.07	(0.75; 1.53)	0.89	(0.63; 1.25)
		Upper	ref.	1.06	(0.74; 1.51)	0.85	(0.60; 1.22)
	‘Fruit & vegetables’	Bottom	ref.	ref.		ref.	
		Middle	ref.	1.06	(0.76; 1.49)	1.32	(0.94; 1.87)
		Upper	ref.	1.67**	(1.17; 2.38)	2.47****	(1.73; 3.54)
	‘Fast food & sweets’	Bottom	ref.	ref.		ref.	
		Middle	ref.	1.04	(0.73; 1.46)	0.83	(0.58; 1.17)
		Upper	ref.	0.99	(0.70; 1.39)	0.95	(0.68; 1.34)
	‘Dairy & fats’	Bottom	ref.	ref.		ref.	
		Middle	ref.	1.06	(0.75; 1.50)	1.28	(0.90; 1.81)
		Upper	ref.	1.43*	(1.01; 2.00)	1.38	(0.97; 1.96)

All data adjusted for sample weights. Odd Ratios were further adjusted for age (continuous variable), BMI (categorical variable) and socioeconomic status (continuous variable measured as SES index which was calculated from four single components: mother’s education, father’s education, economic status, description of household)

*p < 0.05, **p < 0.01, ***p < 0.001, ****p < 0.0001

## Discussion

Our study revealed that in Polish girls physical activity at school/work, during recreation time and related to work at home’s yard was positively associated with the frequency of fruit and vegetable (FV) consumption. Furthermore, we found inverse associations between certain patterns of physical activity and unhealthy dietary behaviours. An unexpected finding was that school/work related activity contributed to higher levels of physical activity than active recreation or any other activities. The present study failed to find associations between 'Walking and domestic activity' physical activity pattern and any of the dietary patterns.

Girls particularly active at school were more likely to consume FV more often than girls whose activity was not school/work related. The coexisting behaviours found in our study can be related to personal or environmental factors. It has to be made clear, that girls with the highest adherence to the 'School/work activity' pattern were not girls who simply attended school. Consistently with the national sociodemographic data [47], over 90 % of our study participants declared being in primary, secondary or tertiary education, with compulsory participation in 3 to 4 h of Physical Education (PE) a week [39]. This suggest that girls who adhered to 'School/work activity' pattern may have had specific personality traits, e.g., self-efficacy, which was the motive to get more involved in activities provided by the school than their peers [48]. Interestingly, the same personality traits are linked with better eating habits in adolescent girls [49, 50]. Highly motivated students [51], those who spend more time studying [52], or those with high academic performance [53], have shown to adopt healthier behaviours in comparison to non-achievers [53–56]. Apart from the personal dimension, environmental factors cannot be underestimated. Health-orientated schools, with better provision of PE, better facilities and perhaps more developed healthy eating policies may have had influenced the coexisting behaviours [57]. Nevertheless, our finding suggests that there might be a common denominator for associated behaviours in adolescent girls who choose to be active at school, worth further investigation.

Similarly to girls mainly active at school, girls who chose to be active in their leisure time, had increased likelihood of frequent FV consumption. Additionally, they have shown to be less likely to follow traditional Polish diet, characterised by starchy and high fat foods. Participation in extracurricular sport activities complemented by healthy dietary behaviours suggests that girls with the highest adherence to the 'Active recreation' pattern made conscious lifestyle choices. The association between active lifestyle and healthy diet was previously well documented in adult females [58, 59], but associations in adolescent girls are not clear [60]. Perhaps, media messages to ‘look and feel good’, had a stronger impact on Polish girls, resulting in emulating adult behaviours at young age.

Healthy dietary behaviours were also observed among girls with the highest adherence to the 'Yard activity' pattern, characterised mainly by garden maintenance. Girls with the predominance of this type of physical activity were more likely to have higher frequency of FV consumption and less likely to adhere to 'Fast foods and sweets' dietary patterns. Being involved in house chores may be an indicator of authoritative parenting style which has been previously associated with dietary intake [61]. Furthermore, our study is in accordance with studies that evaluated the effectiveness of school/community garden interventions [62–64]. Students who participated in gardening programs reported higher FV intakes [62, 63] and lower fast foods consumption to their peers who did not take part in the interventions [64]. Although the setting in our study relates to home yards, the mechanisms behind gardening and healthy dietary choices may be of a similar nature. On the other hand, some studies are sceptical about the effectiveness of these types of intervention in youth [65].

An interesting finding was, that girls mostly active at school or work had the highest levels of overall physical activity in comparison to girls with different patterns of activity. Perhaps, necessity to engage in physical activity is more effective in adolescent girls than personal choice. High level of physical activity among girls active at school have been previously observed by Boone-Heinonen [13]. In his study, 'School Clubs & Sports' was the only high physical activity cluster in adolescent females. Although he did not find any associations between this pattern and the diet, he discovered that girls active at school had the lowest rates of obesity [13]. These are promising findings, suggesting that school may be an effective setting for implementing healthy lifestyle strategies.

We did not find any clear associations between the 'Walking and domestic activity' pattern and dietary behaviours. Most previous studies associated sedentary physical activity patterns with unhealthy diets in adolescents [14, 31]. However, the pattern found in our study was not strictly sedentary and apart from sitting, consisted of house chores and walking. Surprisingly, girls with the predominance of this type of activity had relatively high overall physical activity, which can be explained by the cumulative effect of all the activities. It could be, that the variety of different activities within this pattern did not allow to link it with any of the specific dietary patterns.

### Strengths

One of the major strengths of this study is a large, nationally representative sample of 1107 girls. Although our findings are specific to Polish population (of females only) and should not be generalised to the wide population of adolescents, our study provides a valuable insight into the behaviours of adolescent girls from a country that in many aspects is becoming increasingly westernised [9]. Also, to our knowledge this was the first study which used PCA to derive two groups of patterns (physical activity and dietary) separately, and examined the associations using logistic regression in a population of adolescent girls. To address robustness of our results, odds ratios were adjusted for potential confounders. Finally, the total variance explained for each group of patterns was relatively high, i.e., 52.2 % for physical activity patterns and 33.9 % for dietary patterns, which in comparison to previous studies [66] is another asset of the study.

### Limitations

The limitations of our study relate predominantly to the potential biases that may occur when self-reported data is analysed. First, we used a subjective method of measuring physical activity. As evidence suggests, self-reported intensity of physical activity in overweight adolescents tends to be often overestimated [67]. The use of accelerometers combined with self-reported data could probably provide more accurate information, as suggested by Jago [68]. Also, as shown in a systematic review by Lee et al. [69], short version of IPAQ can significantly overestimate physical activity level, but there is no detailed information regarding long version of IPAQ. The validity and reliability of long version of IPAQ has been tested by Craig et al. [70] in the 12 countries study and is a tool that has been successfully used to assess levels of physical activity among Polish adolescents [71]. Thus, we decided to use the long form of IPAQ as a more feasible method in a large representative sample, with less of a burden upon participants.

Secondly, it could be argued that the use of FFQs often leads to overestimation of some foods consumption and can be less accurate in estimation of daily intakes, in comparison to other methods of dietary assessment [72, 73]. It has been well documented, that similarly to adult populations, young people often overestimate the consumption of foods perceived as healthy (particularly FV) and misreport on the consumption of unhealthy foods, such as fast foods, snacks and sugary drink [74]. However, we have chosen FFQ because we aimed to screen predominantly for 'healthy' and 'unhealthy' dietary behaviours, rather than the exact amounts of consumed foods. Therefore, we decided that the use of two Block questionnaires complemented by our Food Variety Questionnaire (all previously validated in Polish population) was the approach that fitted better with the aim of our study.

Finally, despite many advantages of PCA, data driven methods require researchers' subjective decisions. Thus, the results of our study need to be carefully compared with the results from other studies.

## Conclusions

We found associations between physical activity patterns and dietary patterns in Polish girls, which suggests that common denominators for such behaviours may exist in this population. Girls with the highest adherence to the ‘School/work activity’ pattern had the highest levels of physical activity among all other physical activity patterns and presented pro-healthy dietary behaviours. Therefore, school should be recognised as an important setting to maximise girls' physical activity potential. The adherence to ‘Active recreation’ and ‘Yard activity’ patterns did not result in the highest levels of physical activity, but was associated with healthy dietary behaviours. Therefore, the 'after-school' time is the area that should also be targeted to increase the overall daily physical activity. Acquiring a habit of being active outside school or work may help to sustain the levels of physical activity after completing education. Future studies could investigate if the associations found in our study were only country specific, what is a potential common denominator for such clustering of behaviours and what are the health outcomes of associated behaviours.

## Abbreviations

BMI, body mass index; BSQF, Block Screening Questionnaire for Fat Intake; BSQFVF, Block Screening Questionnaire for Fruit/Vegetable/Fibre Intake; CI, confidence interval; FIVeQ, Food Intake Variety Questionnaire; FV, fruit and vegetables; GEBaHealth, Girls Eating Behaviours and Health; IOTF, International Obesity Task Force; IPAQ-L, International Physical Activity Questionnaire – Long; IPAQ-S, International Physical Activity Questionnaire – Short; KMO, Kaiser-Meyer-Olkin; MET, metabolic energy turnover; OR, odds ratio; PA, physical activity; PCA, principal component analysis; SES, socioeconomic status

## Additional files

Additional file 1: Table S1. Sample characteristics: school and work status. (DOCX 1.8 kb) Additional file 2: Table S2. Factor-loading matrix for the 4 major physical activity patterns identyfied by principal component analysis: after excluding from the analysis of 47 girls who were solely in employment (*n* = 1060). (DOCX 16kb) Additional file 3: Table S3. Means with 95 % CI for dietary characteristics and physical activities. (DOCX 19 kb) Additional file 4: Table S4. Unadjusted associations between physical activity and dietary patterns (unadjusted odd ratios with 95 % CI). (DOCX 17 kb)
