# Supplementary material for: Associations between physical activity patterns and dietary patterns in a representative sample of Polish girls aged 13-21 years: a cross-sectional study (GEBaHealth Project)
Source: BMC Public Health. 2016 Aug 2;16:698. doi: 10.1186/s12889-016-3367-4 (PMC4971681; doi:10.1186/s12889-016-3367-4)
Supplement: Additional file 1: Table S1. — Sample characteristics: school and work status. (DOCX 1.8 kb) [file 12889_2016_3367_MOESM1_ESM.docx]

**Additional file 1: Table S1. Sample characteristics: school and work status**

| GEBaHealth (n=1107) | Sample (n) | % |
| --- | --- | --- |
| Studying | 932 | 84.2 |
| Working and studying | 74 | 6.7 |
| Working | 47 | 4.2 |
| Not working and not studying | 54 | 4.9 |
