# Supplementary material for: Associations between physical activity patterns and dietary patterns in a representative sample of Polish girls aged 13-21 years: a cross-sectional study (GEBaHealth Project)
Source: BMC Public Health. 2016 Aug 2;16:698. doi: 10.1186/s12889-016-3367-4 (PMC4971681; doi:10.1186/s12889-016-3367-4)
Supplement: Additional file 2: Table S2. — Factor-loading matrix for the 4 major physical activity patterns identyfied by principal component analysis: after excluding from the analysis of 47 girls who were solely in employment (n = 1060). (DOCX 16kb) [file 12889_2016_3367_MOESM2_ESM.docx]

**Additional file 2: Table S2.** Factor-loading matrix for the 4 major physical activity patterns identified by principal component analysis: after excluding from the analysis of 47 girls who were solely in employment (n=1,060)

| Type of physical activity | Factor 1 | Factor 2 | Factor 3 | Factor 4 |
| --- | --- | --- | --- | --- |
|  | ‘Active recreation’ | ‘Yard activity’ | ‘School/work activity’ | ‘Walking & domestic activity’ |
| School/work – moderate activity |  |  | 0.78 |  |
| School/work – walking |  |  | 0.68 |  |
| School/work – vigorous activity |  |  | 0.76 |  |
| Leisure-time – moderate activity | 0.80 |  |  |  |
| Leisure-time – vigorous activity | 0.72 |  |  |  |
| Active transportation – cycle | 0.62 |  |  |  |
| Yard work – vigorous activity |  | 0.77 |  |  |
| Yard work – moderate activity |  | 0.80 |  |  |
| Active transportation – walking |  |  |  | 0.73 |
| Leisure-time – walking |  |  |  | 0.61 |
| Home activity – moderate |  |  |  | 0.52 |
| Sitting |  |  |  |  |
| Factor intercorrelations |  |  |  |  |
| Factor 1 (Active recreation) | -- |  |  |  |
| Factor 2 (Yard activity) | 0.13 | -- |  |  |
| Factor 3 (School/work activity) | 0.17 | 0.08 | -- |  |
| Factor 4 (Walking & domestic activity) | 0.07 | 0.12 | 0.14 | -- |
| Eigenvalues | 2.16 | 1.50 | 1.47 | 1.22 |
| Variance explained (%)^b^ | 18.0 | 12.5 | 12.2 | 10.2 |

Notes: Factor loadings of ≤ |0.50| are not shown in the table for simplicity. Factors are not sorted by loadings (order of the factors refers to the one presented in the original manuscript, to ease the comparison). All data adjusted for sample weights.

^a^ Physical activity was expressed in MET-minutes/week.

^b^ Total variance in physical activity variables explained by 4 patterns is 52.9%.
