# Supplementary material for: Associations between physical activity patterns and dietary patterns in a representative sample of Polish girls aged 13-21 years: a cross-sectional study (GEBaHealth Project)
Source: BMC Public Health. 2016 Aug 2;16:698. doi: 10.1186/s12889-016-3367-4 (PMC4971681; doi:10.1186/s12889-016-3367-4)
Supplement: Additional file 3: Table S3. — Means with 95 % CI for dietary characteristics and physical activities. (DOCX 19 kb) [file 12889_2016_3367_MOESM3_ESM.docx]

**Additional file 3: Table S3.** Means with 95% Confidence Intervals (95%CI) for dietary characteristics and physical activities

|  |  | Mean | (95%CI) |
| --- | --- | --- | --- |
|  |  |  |  |
| Food frequency consumption of (points): | Fruit or vegetable juices^a^ | 1.7 | (1.6; 1.8) |
|  | Fruit (without juices)^a^ | 2.5 | (2.4; 2.6) |
|  | Green salad^a^ | 2.1 | (2.0; 2.1) |
|  | Potatoes^a^ | 2.7 | (2.6; 2.7) |
|  | Beans^a^ | 0.7 | (0.7; 0.8) |
|  | Prepared vegetables^a^ | 1.7 | (1.6; 1.8) |
|  | High-fiber or bran cereal^a^ | 1.1 | (1.0; 1.2) |
|  | Wholegrain bread^a^ | 1.5 | (1.4; 1.6) |
|  | White bread (including biscuits, muffins)^a^ | 3.1 | (3.0; 3.1) |
|  | Hamburgers or cheeseburgers^b^ | 0.3 | (0.3; 0.4) |
|  | Red meats^b^ | 1.3 | (1.2; 1.3) |
|  | Fried chicken^b^ | 2.0 | (1.9; 2.0) |
|  | Hot dogs or frankfurters^b^ | 1.2 | (1.1; 1.3) |
|  | Luncheon meats or bacon or fatty sausages^b^ | 0.5 | (0.5; 0.6) |
|  | Salad dressings or mayonnaise (not diet)^b^ | 1.4 | (1.3; 1.4) |
|  | Margarine or butter^b^ | 3.4 | (3.4; 3.5) |
|  | Eggs^b^ | 2.0 | (2.0; 2.1) |
|  | Cheese or cheese spread^b^ | 2.5 | (2.4; 2.5) |
|  | Whole milk^b^ | 1.9 | (1.8; 2.0) |
|  | French fries or potato chips or corn chips or popcorn^b^ | 1.3 | (1.2; 1.3) |
|  | Ice cream^b^ | 0.6 | (0.5; 0.6) |
|  | Doughnuts or pastries or cake or cookies^b^ | 1.9 | (1.8; 2.0) |
| Food intake variety by food groups (foods/week): | Cereals and potatoes^c^ | 3.7 | (3.7; 3.8) |
|  | Dairy products^c^ | 2.3 | (2.3; 2.4) |
|  | Meats, fish and eggs^c^ | 4.4 | (4.3; 4.5) |
|  | Vegetables^c^ | 7.2 | (7.1; 7.4) |
|  | Fruit^c^ | 3.6 | (3.5; 3.7) |
|  | Fats^c^ | 3.5 | (3.4; 3.6) |
|  | Sweets and snacks^c^ | 3.1 | (3.0; 3.1) |
|  | Beverages (without alcohols)^c^ | 3.5 | (3.4; 3.6) |
| Physical activity (MET-minutes/week): | School/work – walking | 182 | (167; 197) |
|  | School/work – moderate activity | 105 | (95; 115) |
|  | School/work – vigorous activity | 219 | (196; 242) |
|  | Leisure-time – walking | 70 | (62; 77) |
|  | Leisure-time – moderate activity | 27 | (23; 30) |
|  | Leisure-time – vigorous activity | 53 | (45; 61) |
|  | Active transportation – walking | 143 | (131; 154) |
|  | Active transportation – cycle | 22 | (17; 28) |
|  | Yard work – moderate activity | 33 | (29; 37) |
|  | Yard work – vigorous activity | 28 | (22; 33) |
|  | Home activity – moderate | 70 | (65; 76) |
|  | Sitting | 78 | (73; 82) |

Notes: All data adjusted for sample weights.

^a^ For 9 food items the consumption frequency categories were coded as numerical data: less than once per week (0 points), about once per week (1 point), 2-3 times per week (2 points), 4-6 times per week (3 points), daily (4 points).

^b^ For 13 food items the consumption frequency categories were coded as numerical data: less than once per month (0 points), 2-3 times per month (1 point), 1-2 times per week (2 points), 3-4 times per week (3 points), 5 times per week and more (4 points).

^c^ Ranges of food intake variety by food groups were: cereals and potatoes: 0-6 foods/week, dairy products: 0-4 foods/week, meats, fish and eggs: 0-12 foods/week, vegetables: 0-14 foods/week, fruit: 0-8 foods/week, fats: 0-6 foods/week, sweets and snacks: 0-4 foods/week, beverages (without alcohols): 0-6 foods/week.
