# Supplementary material for: Associations between physical activity patterns and dietary patterns in a representative sample of Polish girls aged 13-21 years: a cross-sectional study (GEBaHealth Project)
Source: BMC Public Health. 2016 Aug 2;16:698. doi: 10.1186/s12889-016-3367-4 (PMC4971681; doi:10.1186/s12889-016-3367-4)
Supplement: Additional file 4: Table S4. — Unadjusted associations between physical activity and dietary patterns (unadjusted odd ratios with 95 % CI). (DOCX 17 kb) [file 12889_2016_3367_MOESM4_ESM.docx]

**Additional file 4: Table S4.** Unadjusted associations between physical activity patterns and dietary patterns (Unadjusted Odds Ratios with 95 % Confidence Intervals)

| Physical activity patterns | Dietary patterns | Tertiles of dietary patterns | Tertiles of physical activity patterns | | | | |
| --- | --- | --- | --- | --- | --- | --- | --- |
|  |  |  | Bottom | Middle | | Upper | |
| ‘School/work activity’ | ‘Traditional Polish’ | Bottom | ref. | ref. |  | ref. |  |
|  |  | Middle | ref. | 1.05 | (0.77; 1.44) | 0.95 | (0.68; 1.33) |
|  |  | Upper | ref. | 1.04 | (0.68; 1.57) | 0.99 | (0.72; 1.36) |
|  | ‘Fruit & vegetables’’ | Bottom | ref. | ref. |  | ref. |  |
|  |  | Middle | ref. | 1.18 | (0.85; 1.63) | 1.28 | (0.92; 1.80) |
|  |  | Upper | ref. | 1.47* | (1.05; 2.06) | 2.00**** | (1.43; 2.81) |
|  | ‘Fast food & sweets’ | Bottom | ref. | ref. |  | ref. |  |
|  |  | Middle | ref. | 1.27 | (0.90; 1.78) | 0.84 | (0.60; 1.18) |
|  |  | Upper | ref. | 1.29 | (0.92; 1.81) | 1.05 | (0.75; 1.46) |
|  | ‘Dairy & fats’ | Bottom | ref. | ref. |  | ref. |  |
|  |  | Middle | ref. | 1.13 | (0.81; 1.59) | 1.15 | (0.83; 1.61) |
|  |  | Upper | ref. | 1.57** | (1.12; 2.19) | 1.35 | (0.96; 1.88) |
| ‘Active recreation’ | ‘Traditional Polish’ | Bottom | ref. | ref. |  | ref. |  |
|  |  | Middle | ref. | 0.63* | (0.45-0.90) | 0.67* | (0.48; 0.94) |
|  |  | Upper | ref. | 0.66* | (0.47-0.92) | 0.51*** | (0.36; 0.72) |
|  | ‘Fruit & vegetables’’ | Bottom | ref. | ref. |  | ref. |  |
|  |  | Middle | ref. | 1.43* | (1.03-1.99) | 1.78*** | (1.26; 2.50) |
|  |  | Upper | ref. | 1.19 | (0.85-1.66) | 1.96**** | (1.40; 2.74) |
|  | ‘Fast food & sweets’ | Bottom | ref. | ref. |  | ref. |  |
|  |  | Middle | ref. | 1.13 | (0.80-1.59) | 0.89 | (0.63; 1.25) |
|  |  | Upper | ref. | 0.83 | (0.59; 1.16) | 0.73 | (0.52; 1.01) |
|  | ‘Dairy & fats’ | Bottom | ref. | ref. |  | ref. |  |
|  |  | Middle | ref. | 0.96 | (0.69; 1.35) | 1.08 | (0.77; 1.51) |
|  |  | Upper | ref. | 1.13 | (0.81; 1.59) | 1.37 | (0.99; 1.92) |
| ‘Yard activity’ pattern | ‘Traditional Polish’ | Bottom | ref. | ref. |  | ref. |  |
|  |  | Middle | ref. | 0.72 | (0.52; 1.01) | 0.90 | (0.64; 1.27) |
|  |  | Upper | ref. | 0.82 | (0.59; 1.15) | 0.91 | (0.65; 1.28) |
|  | ‘Fruit & vegetables’ | Bottom | ref. | ref. |  | ref. |  |
|  |  | Middle | ref. | 1.24 | (0.90; 1.73) | 1.70** | (1.21; 2.39) |
|  |  | Upper | ref. | 1.36 | (0.97; 1.90) | 2.15**** | (1.53; 3.03) |
|  | ‘Fast food & sweets’ | Bottom | ref. | ref. |  | ref. |  |
|  |  | Middle | ref. | 0.68* | (0.48; 0.96) | 0.68* | (0.48; 0.96) |
|  |  | Upper | ref. | 0.61** | (0.44; 0.86) | 0.51**** | (0.36; 0.71) |
|  | ‘Dairy & fats’ | Bottom | ref. | ref. |  | ref. |  |
|  |  | Middle | ref. | 0.79 | (0.57; 1.11) | 0.92 | (0.66; 1.30) |
|  |  | Upper | ref. | 1.02 | (0.72; 1.46) | 1.10 | (0.78; 1.54) |
| ‘Walking & domestic activity’ | ‘Traditional Polish’ | Bottom | ref. | ref. |  | ref. |  |
|  |  | Middle | ref. | 1.27 | (0.91; 1.77) | 1.30 | (0.93; 1.82) |
|  |  | Upper | ref. | 1.17 | (0.84; 1.63) | 1.32 | (0.95; 1.84) |
|  | ‘Fruit & vegetables’ | Bottom | ref. | ref. |  | ref. |  |
|  |  | Middle | ref. | 0.90 | (0.64; 1.26) | 0.91 | (0.66; 1.27) |
|  |  | Upper | ref. | 1.01 | (0.61; 1.67) | 0.86 | (0.62; 1.21) |
|  | ‘Fast food & sweets’ | Bottom | ref. | ref. |  | ref. |  |
|  |  | Middle | ref. | 1.25 | (0.89; 1.76) | 1.07 | (0.75; 1.52) |
|  |  | Upper | ref. | 1.23 | (0.88; 1.72) | 0.94 | (0.67; 1.31) |
|  | ‘Dairy & fats’ | Bottom | ref. | ref. |  | ref. |  |
|  |  | Middle | ref. | 0.78 | (0.56; 1.10) | 0.97 | (0.69; 1.38) |
|  |  | Upper | ref. | 0.91 | (0.65; 1.26) | 1.03 | (0.73; 1.45) |
| Total physical activity | ‘Traditional Polish’ | Bottom | ref. | ref. |  | ref. |  |
|  |  | Middle | ref. | 1.02 | (0.73; 1.43) | 0.86 | (0.61; 1.20) |
|  |  | Upper | ref. | 0.94 | (0.67; 1.32) | 0.86 | (0.61; 1.20) |
|  | ‘Fruit & vegetables’ | Bottom | ref. | ref. |  | ref. |  |
|  |  | Middle | ref. | 1.11 | (0.80; 1.53) | 1.37 | (0.98; 1.92) |
|  |  | Upper | ref. | 1.76** | (1.25; 2.48) | 2.67**** | (1.89; 3.78) |
|  | ‘Fast food & sweets’ | Bottom | ref. | ref. |  | ref. |  |
|  |  | Middle | ref. | 1.04 | (0.78; 1.39) | 0.82 | (0.59; 1.15) |
|  |  | Upper | ref. | 0.98 | (0.69; 1.40) | 0.90 | (0.64; 1.25) |
|  | ‘Dairy & fats’ | Bottom | ref. | ref. |  | ref. |  |
|  |  | Middle | ref. | 1.09 | (0.77; 1.53) | 1.18 | (0.84; 1.65) |
|  |  | Upper | ref. | 1.45* | (1.04; 2.02) | 1.34 | (0.96; 1.87) |

Notes: All data adjusted for sample weights.

**p*<0.05, ***p*<0.01, ****p*<0.001, *****p*<0.0001.
